# No prognostic value added by vitamin D pathway SNPs to current prognostic system for melanoma survival

**DOI:** 10.1371/journal.pone.0174234

**Published:** 2017-03-21

**Authors:** Li Luo, Irene Orlow, Peter A. Kanetsky, Nancy E. Thomas, Shenying Fang, Jeffrey E. Lee, Marianne Berwick, Ji-Hyun Lee, on behalf of the GEM Study Group

**Affiliations:** 1 Department of Internal Medicine, University of New Mexico, Albuquerque, New Mexico, United States of America; 2 University of New Mexico Comprehensive Cancer Center, Albuquerque, New Mexico, United States of America; 3 Department of Epidemiology and Biostatistics, Epidemiology Service, Memorial Sloan Kettering Cancer Center, New York, New York, United States of America; 4 Department of Cancer Epidemiology, H. Lee Moffitt Cancer Center & Research Institute, Tampa, Florida, United States of America; 5 Department of Dermatology, University of North Carolina, Chapel Hill, North Carolina, United States of America; 6 Department of Surgical Oncology, The University of Texas MD Anderson Cancer Center, Houston, Texas, United States of America; University of Alabama at Birmingham, UNITED STATES

## Abstract

The prognostic improvement attributed to genetic markers over current prognostic system has not been well studied for melanoma. The goal of this study is to evaluate the added prognostic value of Vitamin D Pathway (VitD) SNPs to currently known clinical and demographic factors such as age, sex, Breslow thickness, mitosis and ulceration (CDF). We utilized two large independent well-characterized melanoma studies: the Genes, Environment, and Melanoma (GEM) and MD Anderson studies, and performed variable selection of VitD pathway SNPs and CDF using Random Survival Forest (RSF) method in addition to Cox proportional hazards models. The Harrell’s C-index was used to compare the performance of model predictability. The population-based GEM study enrolled 3,578 incident cases of cutaneous melanoma (CM), and the hospital-based MD Anderson study consisted of 1,804 CM patients. Including both VitD SNPs and CDF yielded C-index of 0.85, which provided slight but not significant improvement by CDF alone (C-index = 0.83) in the GEM study. Similar results were observed in the independent MD Anderson study (C-index = 0.84 and 0.83, respectively). The Cox model identified no significant associations after adjusting for multiplicity. Our results do not support clinically significant prognostic improvements attributable to VitD pathway SNPs over current prognostic system for melanoma survival.

## Introduction

Cutaneous melanoma is a potentially fatal form of skin cancer. More than 10,000 individuals in the US are expected to die from this disease in 2016 [1]. The American Joint Committee on Cancer (AJCC) developed the I-IV staging system [2] for melanoma based on tumor characteristics, including Breslow thickness, ulceration and mitoses, which is the major prognostic classification for melanoma. However, there is a broad range of survival rates among patients of the same stage, and this variability suggests the need for developing new prognostic markers.

Recent developments in genome technologies have provided promise and potential in utilizing germline genetic alterations to develop improved classifications of melanoma leading to more precise prognostication of disease. Current molecular melanoma research has mainly focused on investigating the genetic association with the development and survival of melanoma to gain insights into melanoma etiology and progression, respectively. However, the prognostic improvement attributed to genetic markers over existing clinical and demographic factors has not been well studied. The single nucleotide polymorphisms (SNPs) in the VitD pathway genes have been implicated in the development, progression and survival of melanoma [3, 4]. As well, an interaction effect between the *BsmI* (rs1544410) A allele in the *VDR* gene and serum vitamin D levels on outcome has been reported, and this was despite the lack of observed main effect of *VDR* genotypes on outcome [5]. Another study demonstrated an association between vitamin D deficiency at time of diagnosis and thicker melanoma, a marker of poorer prognosis [6].

The objective of this study is to evaluate whether incorporation of SNPs in the VitD pathway would provide improved melanoma prognostic classification. Specifically, we investigated whether coupling VitD pathway SNPs with known clinical and demographic information provides better prognostic effects than clinical/demographic factors alone for melanoma specific survival, in an international population based genetic epidemiology of melanoma, the Genes, Environment, and Melanoma (GEM) study. We used the genotype and phenotype data from the MD Anderson study as an independent validation [7].

## Materials and methods

The study design, sample and data collection methods for the GEM study were described elsewhere [8]. In brief, the GEM Study is an international population-based study of melanoma development and progression consisting of 3,578 incident cases of cutaneous melanoma, where controls (n = 2,372) were newly diagnosed with invasive single primary melanomas and the cases (n = 1,206) were newly diagnosed with multiple primary melanomas. The sample collection, germline buccal DNA extraction, and genotyping pipeline along with standard quality control procedures for the 38 *VDR* SNPs in GEM using the Sequenom MassARRAY iPLEX platform, pyrosequencing and melting temperature assays were previously described [3, 9]. A subset of SNPs were genotyped using a custom Illumina GoldenGate assay, where standard quality control procedures were performed to ensure the quality of the Illumina SNP data. Specifically, we visually evaluated the genotype clustering images and excluded samples and SNPs with genotyping call rates of <90%. There are 2993 samples genotyped using Illumina that passed quality control. Assays were considered optimal according to degree of clustering of repeats, the absence of signal in controls and reproducibility. Data on major clinical prognostic factors for melanoma, demographic, sun exposure and histopathological variables were collected for all study participants and were described previously [10]. Seventy SNPs from genes in the VitD pathway, including 38 *VDR* SNPs and 32 additional SNPs genotyped by Sequenom or Illumina platform, among 3,566 white subjects were included in the analyses. To illustrate the genes evaluated in this study along with their biological roles, we have included a vitamin D pathway diagram (S1 Fig) adapted from a previous report [11] which also incorporates the anti-cancer effects of vitamin D [12, 13]. The list of SNPs along with their chromosome locations, minor/major alleles in the population, gene names, minor allele frequency (MAF) in the GEM study participants, and genotyping platform are shown in S1 Table. These include functionally relevant SNPs or SNPs previously reported in other genetic association studies that included the Vitamin D pathway genes [9, 11]. The linkage disequilibrium pattern for the 38 VDR SNPs was described elsewhere [9].

The MD Anderson study is a hospital-based study of cutaneous melanoma, consisting of 1804 melanoma patients presenting to clinics at MD Anderson [7]. A total of 1788 samples among them were successfully genotyped and passed quality control using the Illumina Human Omni1-Quad_v1-0_B array. The sample collection, DNA extraction methods, genotyping platform, and standard quality control procedures were described previously [7]. Genome-wide genotyping imputation was conducted using MACH [14] and the Hapmap2 CEU population reference panel [7]. Among the 70 VitD pathway SNPs genotyped in GEM, 65 were either genotyped or imputed in the MD Anderson study, and were evaluated as an independent validation. The genotype data from the MD Anderson study [7] was obtained from dbGap database (http://www.ncbi.nlm.nih.gov/gap) with accession number: phs000187.v1.p1. The phenotype data and follow-up survival outcome from the MD Anderson study was supplied by the study investigators.

The study protocol was approved by the Institutional Review Boards of all participating institutions, including those at the British Columbia Cancer Agency, Vancouver, BC, CA; Cancer Care Ontario, Toronto, ON, CA; Centro per la Prevenzione Oncologia, Torino, IT; Memorial Sloan Kettering Cancer Center, New York, NY, US; Menzies Cancer Center, Hobart, TAS, AU; University of California, Irvine, CA, US; University of Michigan, Ann Arbor, MI, US; University of North Carolina, Chapel Hill, NC, US; University of Sydney, Sydney, NSW, AU, and the University of Texas MD Anderson Cancer Center, Houston, TX, US. Written informed consent was obtained from each participant.

Utilizing the genotype and phenotype data from the GEM study and the MD Anderson study, we investigated the prognostic improvement of VitD pathway SNPs over the major known prognostic factors including: age, sex, Breslow thickness, mitoses and ulceration. In the GEM study we also performed a secondary analysis by including other additional prognostic factors (histology, site, sun exposure, and phenotypic index) to evaluate the change in results and model performance.

Summary statistics were used to describe the patient demographics and characteristics of the common clinical and demographical factors in the two studies. Two-sided t-tests and Wilcoxon rank-sum tests were used to compare continuous variables. Chi-squared tests were performed to compare categorical variables. We applied the Random Survival Forest (RSF) method [15] as well as Cox proportional hazards models to data from the two melanoma studies. RSF is an ensemble tree method for analysis of right censored survival data [15]. Each tree is built using a recursive portioning method to split the feature space, spanned by all predictor variables, into groups of subjects with similar association patterns between the predictor variables and the survival outcome. Specifically, each tree is grown using a randomly drawn bootstrap sample of the data. Based on a randomly selected subset of the variables, a survival criterion involving survival time and censoring status information (log-rank test) is used to split the tree nodes. Prediction is made by averaging over an ensemble of trees. Important variables were selected based on two measures of the predictiveness of variables in a tree: 1) variable importance (VIMP) [15], and 2) minimal depth of maximal subtree [16]. VIMP is a measure of how important a variable is, which estimates the change in prediction error if that variable is eliminated from analysis. Minimal depth assesses the predictiveness of variables in a tree by estimating the minimal depth relative to the root node. A larger VIMP value or a smaller minimal depth corresponds to better predictiveness of a variable.

For Cox proportional hazard regression models, Harrell’s C-index, an extension of the area under the ROC curve (AUC), was calculated to measure the concordance probability between prognostic factors and survival outcome and thus compare the predictability of the selected variables.

To further evaluate the prognostic effects of VitD SNPs on melanoma survival, we examined the relationship between VitD pathway SNPs and Breslow thickness as a marker of melanoma prognosis. Linear regression models were performed to assess the association between SNPs and log transformed Breslow thicknesses. We also estimated the average causal mediation effect using a quasi-Bayesian Monte Carlo causal mediation analysis method [17, 18] to investigate the impact of each SNP on Melanoma prognosis mediated by Breslow thickness. Multiple comparisons were adjusted using Benjamini and Hochberg (BH) false discovery rate procedure [19].

## Results

The patient demographics and characteristics are displayed in Table 1. Compared to the subjects in GEM, patients in the MD Anderson study were younger, had thicker tumors, more ulceration, more mitoses, and more melanoma deaths. The gender distributions were similar in the two studies. The differences between the two studies suggested more aggressive tumors in the hospital-based MD Anderson study compared to the population-based GEM study.

**Table 1:** Patient demographics and characteristics of the GEM and MD Anderson studies.

Variable	GEM Study (N = 3566)	MD Anderson Study (N = 1788)	P-Value
**AgeMean ± SD (N)**	58.2 ± 15.9 (N = 3566)	52.0 ± 14.5 (N = 1788)	<.0001^a^
**Median (min-max)**	59.0 (7.0-97.0)	52.3 (14.9-94.1)	
**Log Breslow**			
**Mean ± SD (N)**	-0.1 ± 0.8 (N = 3480)	0.2 ± 0.9 (N = 1536)	<.0001^a^
**Median (min-max)**	-0.3 (-3.2 to 3.4)	0.1 (-2.3 to 3.6)	
**Follow up in months Mean ± SD (N)**	83.6 ± 18.7 (N = 3566)	91.1 ± 58.7 (N = 1786)	0.025^b^
**Median (min-max)**	91.0 (4.8-127.0)	82.2 (1.6-625.9)	
**Melanoma Specific Death**			
**No**	3312 (92.9%)	1472 (82.3%)	<.0001^c^
**Yes**	254 (7.1%)	316 (17.7%)	
**Sex**			
**M**	2004 (56.2%)	1050 (58.7%)	0.078^c^
**F**	1562 (43.8%)	738 (41.3%)	
**Ulceration**			
**Missing**	829 (23.2%)	371 (20.7%)	<.0001^c^
**Absent**	2475 (69.4%)	1158 (64.8%)	
**Present**	262 (7.3%)	259 (14.5%)	
**Mitoses**			
**missing**	819 (23.0%)	438 (24.5%)	<.0001^c^
**Absent**	1520 (42.6%)	415 (23.2%)	
**Present**	1227 (34.4%)	935 (52.3%)	

^a^ P value was based on the pooled variances t-test.

^b^ P value was based on the Wilcoxon rank-sum test.

^c^ P value was based on the Chi-square test.

The variables with positive VIMP values selected by the RSF method are described in Table 2 in which major prognostic variables (age, sex, Breslow thickness, mitosis, and ulceration) are displayed in italic font and the “rs” (Reference Sequence) numbers denote VitD pathway SNPs. In both the GEM study and MD Anderson study, existing clinical and demographic prognostic factors had larger VIMP values, indicating higher predictability for melanoma survival compared to SNPs. Table 3 gives results of the secondary analyses of including additional prognostic variables (histology, site, sun exposure, and phenotypic index) in the RSF model in the GEM study. We again observed higher prognostic effects of the clinical and demographical factors than the SNPs on melanoma specific survival.

**Table 2:** The major clinical and demographical prognostic variables and genetic variants selected by Random Survival Forest (RSF) ordered based on the variable importance (VIMP) prediction measure.

GEM Study		MD Anderson Study	
Variable	VIMP^a^	Variable	VIMP
*logBreslow*	0.047	*logBreslow*	0.053
*Mitoses*	0.0074	*Ulceration*	0.016
*Ulceration*	0.0069	*Mitoses*	0.0053
*Age*	0.0045	*Age*	0.0028
*Sex*	0.0012	rs7594289 (*CYP27A1*)	0.0018
rs12512631 (*GC*) ^b^	0.00060	*Sex*	0.0014
rs3787555 (*CYP2R1*)	0.00052	rs7861779 (*RXRA*)	0.00054
rs2239182 (*VDR*)	0.00052	rs3782905 (*VDR*)	0.00049
rs7041 (*GC*)	0.00037	rs4646536 (*CYP27B1*)	0.00034
rs10875712 (*VDR*)	0.00036	rs7974708 (*VDR*)	0.00028
rs1051130 (*CCND3*)	0.00033	rs886441 (*VDR*)	0.00027
rs2228570 (*VDR*)	0.00030	rs7299460 (*VDR*)	0.00026
rs2189480 (*VDR*)	0.00029	rs4809959 (*CYP2R1*)	0.00021
rs4760648 (*VDR*)	0.00025	rs11568820 (*VDR*)	0.00019
rs7305032 (*VDR*)	0.00022	rs11168275 (*VDR*)	0.00016
rs2107301 (*VDR*)	0.00019	rs11168287 (*VDR*)	0.00015
rs222040 (*GC*)	0.00019	rs2228570 (*VDR*)	0.00015
rs3218089 (*CCND3*)	0.00017	rs2282679 (*GC*)	0.00014
rs1989969 (*VDR*)	0.00017	rs10875712 (*VDR*)	0.00013
rs7299460 (*VDR*)	0.00013	rs1051130 (*CCND3*)	0.00012
rs927650 (*CYP2R1*)	0.00011	rs2296241 (*CYP2R1*)	0.00010
rs2544027 (*VDR*)	0.000042	rs2189480 (*VDR*)	0.000099
rs10776909 (*RXRA*)	0.000039	rs7041 (*GC*)	0.000079
rs1544410 (*VDR*)	0.000034	rs4760648 (*VDR*)	0.000076
rs2238140 (*VDR*)	0.000033	rs4809960 (*CYP2R1*)	0.000066
rs4760674 (*VDR*)	0.000026	rs2107301 (*VDR*)	0.000048
rs7974708 (*VDR*)	0.000026	rs1790349 (*DHCR7*)	0.000047
rs11574139 (*VDR*)	0.000023	rs4760674 (*VDR*)	0.000032

^a^ Larger VIMP value corresponds to better predictiveness of a variable (see materials and methods section for more details). The clinical, demographical variables and genetic variants selected are ordered based on the VIMP measure.

^b^ SNP gene names are shown in parentheses.

**Table 3:** The clinical, demographical variables and genetic variants selected by Random Survival Forest (RSF) ordered based on the variable importance (VIMP) prediction measure in the secondary analysis of GEM study by including additional prognostic factors.

GEM Study		MD Anderson Study	
Variable	VIMP^a^	Variable	VIMP
*logbreslow*	0.049	*logbreslow*	0.053
*Histology*	0.0054	*Ulceration*	0.016
*Ulceration*	0.0046	*Mitoses*	0.0053
*Age*	0.0030	*Age*	0.0028
*Mitoses*	0.0025	rs7594289 (*CYP27A1*)	0.0018
*Site*	0.0022	*Sex*	0.0014
rs12512631 (*GC*) ^b^	0.00094	rs7861779 (*RXRA*)	0.00054
rs7041	0.00075	rs3782905 (*VDR*)	0.00049
*Burns*	0.00049	rs4646536 (*CYP27B1*)	0.00034
*Status* ^c^	0.00041	rs7974708 (*VDR*)	0.00028
rs1051130 (*CCND3*)	0.00038	rs886441 (*VDR*)	0.00027
*Phenotypic_index*	0.00031	rs7299460 (*VDR*)	0.00026
rs2107301 (*VDR*)	0.00029	rs4809959 (*CYP2R1*)	0.00021
*Freckle*	0.00023	rs11568820 (*VDR*)	0.00019
rs2189480 (*VDR*)	0.00021	rs11168275 (*VDR*)	0.00016
rs2228570 (*VDR*)	0.00020	rs11168287 (*VDR*)	0.00015
*Education*	0.00019	rs2228570 (*VDR*)	0.00015
rs3787555 (*CYP2R1*)	0.00014	rs2282679 (*GC*)	0.00014
rs10776909 (*RXRA*)	0.00012	rs10875712 (*VDR*)	0.00013
rs7974708 (*VDR*)	0.00012	rs1051130 (*CCND3*)	0.00012
rs10875712 (*VDR*)	0.000094	rs2296241 (*CYP2R1*)	0.00010
rs2239182 (*VDR*)	0.000073	rs2189480 (*VDR*)	0.000099
rs7861779 (*RXRA*)	0.000072	rs7041 (*GC*)	0.000079
rs4760648 (*VDR*)	0.000069	rs4760648 (*VDR*)	0.000076
rs927650 (*CYP2R1*)	0.0000078	rs4809960 (*CYP2R1*)	0.000066
rs11168275 (*VDR*)	0.0000035	rs2107301 (*VDR*)	0.000048
		rs1790349 (*DHCR7*)	0.000047
		rs4760674 (*VDR*)	0.000032

^a^ Larger VIMP value corresponds to better predictiveness of a variable (see materials and methods section for more details). The clinical, demographical variables and genetic variants selected are ordered based on the VIMP measure.

^b^ SNP gene names are shown in parentheses.

^c^ Status refers to patients diagnosed with single or multiple primary melanomas.

In the GEM study, including both VitD SNPs and major known prognostic factors selected by the RSF method in a Cox regression model yields a C-index (a concordance measure) of 0.85, which provided slight but not significant improvement by using the known prognostic factors alone (age, sex, Breslow thickness, mitoses and ulceration) alone (C-index = 0.83). Similar results were observed in the MD Anderson study; the C-index is 0.85 for combined SNPs and clinical factors, and 0.84 for clinical factors (age, sex, Breslow thickness, mitoses and ulceration) alone. When additional prognostic factors (i.e. histology, site, sun exposure, and phenotypic index) were included in the analysis of GEM study, we did not observe significant prognostic improvements of incorporating the VitD SNPs (C index = 0.84) over using clinical and demographic factors (age, Breslow thickness, mitoses, ulceration, histology, site, sunburn, status, phenotypic index, freckle, education) alone (C index = 0.83). RSF analyses using minimal depth as the measure of predictiveness yielded similar results (data not shown).

Using the Cox proportional hazards model, we identified nine SNPs nominally significantly associated with melanoma survival in the GEM study (P<0.05), and nine such SNPs in the MD Anderson study (Table 4). Among them the commonly studied rs1544410 (*BsmI*) and rs731236 (*TaqI*) polymorphisms were significant in both studies, suggesting their potential biological role in melanoma survival. However, after correcting for multiple tests, none of the SNPs reached the FDR cutoff of 0.05. Similar results were observed in the analyses of Breslow thickness as a marker for melanoma prognosis in both GEM and MD Anderson study. No significant association between SNPs and Breslow thickness were identified after correcting for multiple tests. The average causal mediation effects measuring the SNP impact on melanoma survival mediated through Breslow thickness yielded no significant results.

**Table 4:** The association between SNPs and melanoma specific survival identified by Cox proportional hazard regression model in the GEM study and MD Anderson study before and after the FDR correction.

		GEM study				MDACC Study				
SNP	genename	N obs	HR (95% CI)^a^	Raw P-value	FDR	N obs	HR (95% CI)^b^	Raw P-value	FDR	type
rs1544410	*VDR*	3401	0.78 (0.65 -0.95)	**0.011**	0.22	1528	0.77 (0.64 -0.94)	**0.008**	0.25	genotyped
rs2239182	*VDR*	3372	1.25 (1.05 -1.48)	**0.012**	0.22	1536	1.17 (0.97 -1.40)	0.10	0.45	genotyped
rs7305032	*VDR*	3250	1.23 (1.02 -1.48)	**0.031**	0.31	1536	0.86 (0.72 -1.04)	0.12	0.45	imputed
rs731236	*VDR*	3406	0.82 (0.68 -0.98)	**0.031**	0.31	1536	0.82 (0.68 -1.00)	**0.046**	0.35	genotyped
rs4760674	*VDR*	3439	1.21 (1.01 -1.44)	**0.038**	0.33	1536	1.07 (0.89 -1.29)	0.48	0.79	genotyped
rs2189480	*VDR*	3414	1.19 (1.00 -1.43)	0.053	0.37	1536	0.95 (0.78 -1.14)	0.56	0.79	genotyped
rs3782905	*VDR*	3445	0.84 (0.69 -1.02)	0.083	0.40	1536	0.91 (0.73 -1.14)	0.42	0.79	imputed
rs2238140	*VDR*	3443	1.17 (0.98 -1.39)	0.09	0.40	1536	0.86 (0.71 -1.04)	0.12	0.45	imputed
rs12370156	*VDR*	3434	1.16 (0.97 -1.38)	0.10	0.40	1536	0.87 (0.72 -1.04)	0.13	0.45	imputed
rs2071358	*VDR*	3427	0.81 (0.63 -1.04)	0.10	0.40	1536	1.07 (0.84 -1.36)	0.60	0.79	genotyped
rs11168284	*VDR*	3414	0.85 (0.70 -1.04)	0.11	0.40	1536	1.15 (0.95 -1.39)	0.16	0.49	imputed
rs7299460	*VDR*	3415	0.86 (0.71 -1.04)	0.12	0.40	1536	1.02 (0.84 -1.25)	0.81	0.93	imputed
rs1989969	*VDR*	3454	1.15 (0.96 -1.38)	0.13	0.40	1536	1.00 (0.83 -1.21)	0.99	0.99	imputed
rs7139166	*VDR*	3376	1.15 (0.96 -1.38)	0.13	0.40	1536	1.04 (0.87 -1.26)	0.65	0.82	imputed
rs4516035	*VDR*	3428	1.15 (0.96 -1.37)	0.13	0.40	1536	1.04 (0.87 -1.26)	0.65	0.82	genotyped
rs7974708	*VDR*	3441	0.87 (0.72 -1.05)	0.14	0.42	1536	0.92 (0.73 -1.17)	0.50	0.79	imputed
rs11168314	*VDR*	3422	0.84 (0.67 -1.06)	0.15	0.42	1536	1.07 (0.84 -1.35)	0.59	0.79	imputed
rs11574139	*VDR*	3445	0.68 (0.39 -1.19)	0.18	0.47	1536	1.15 (0.70 -1.91)	0.58	0.79	imputed
rs1015390	*VDR*	3440	0.83 (0.63 -1.09)	0.18	0.47	1536	0.97 (0.75 -1.26)	0.84	0.93	imputed
rs4073729	*VDR*	3437	0.84 (0.64 -1.10)	0.20	0.50	1536	0.96 (0.74 -1.25)	0.75	0.92	imputed
rs7965281	*VDR*	3261	1.13 (0.93 -1.38)	0.21	0.50	1536	0.90 (0.75 -1.07)	0.22	0.56	imputed
rs10459217	*VDR*	3427	0.87 (0.69 -1.09)	0.22	0.50	1536	0.98 (0.78 -1.25)	0.89	0.93	imputed
rs10875712	*VDR*	3413	1.11 (0.93 -1.33)	0.26	0.54	1536	0.89 (0.74 -1.08)	0.25	0.58	imputed
rs2544027	*VDR*	3447	0.92 (0.77 -1.09)	0.31	0.61	1536	0.94 (0.78 -1.13)	0.51	0.79	imputed
rs11568820	*VDR*	3075	0.89 (0.70 -1.14)	0.35	0.66	1536	1.03 (0.82 -1.29)	0.80	0.93	imputed
rs2107301	*VDR*	3440	1.09 (0.90 -1.32)	0.38	0.70	1531	1.00 (0.81 -1.22)	0.96	0.98	genotyped
rs2544038	*VDR*	3418	0.93 (0.78 -1.10)	0.40	0.70	1536	0.94 (0.78 -1.13)	0.52	0.79	imputed
rs4760648	*VDR*	3429	0.93 (0.77 -1.12)	0.44	0.74	1536	1.18 (0.98 -1.42)	0.079	0.45	genotyped
rs6823	*VDR*	3426	1.07 (0.90 -1.27)	0.47	0.77	1536	0.93 (0.77 -1.12)	0.44	0.79	imputed
rs2544028	*VDR*	3428	0.94 (0.79 -1.12)	0.50	0.78	1536	0.93 (0.77 -1.13)	0.48	0.79	imputed
rs11168275	*VDR*	3463	0.93 (0.75 -1.15)	0.50	0.78	1536	1.06 (0.86 -1.30)	0.58	0.79	genotyped
rs2254210	*VDR*	2810	1.06 (0.87 -1.31)	0.55	0.82	1536	0.83 (0.68 -1.01)	0.064	0.42	genotyped
rs886441	*VDR*	3427	1.07 (0.85 -1.33)	0.58	0.84	1536	1.19 (0.96 -1.48)	0.12	0.45	genotyped
rs4237856	*VDR*	3402	1.04 (0.86 -1.27)	0.67	0.92	1536	1.10 (0.89 -1.36)	0.39	0.77	imputed
rs2228570	*VDR*	3305	0.97 (0.79 -1.18)	0.74	0.93	1536	0.82 (0.69 -0.99)	**0.036**	0.35	imputed
rs2238135	*VDR*	3446	0.97 (0.79 -1.19)	0.75	0.93	1530	0.87 (0.70 -1.08)	0.21	0.55	genotyped
rs10875694	*VDR*	3465	0.97 (0.77 -1.23)	0.80	0.93	1536	0.76 (0.59 -0.98)	**0.032**	0.35	imputed
rs2239181	*VDR*	3437	1.03 (0.76 -1.39)	0.87	0.94	1536	1.20 (0.89 -1.63)	0.24	0.58	genotyped
rs11574143	*VDR*	2780	1.02 (0.73 -1.42)	0.91	0.95	1533	1.25 (0.92 -1.70)	0.15	0.49	genotyped
rs11168287	*VDR*	3411	1.00 (0.83 -1.19)	0.97	0.97	1536	0.88 (0.74 -1.05)	0.17	0.49	genotyped
rs34421776	*TCEAL1*	2733	2.00 (1.16 -3.47)	**0.013**	0.22					N/A^c^
rs10776909	*RXRA*	2724	0.80 (0.63 -1.01)	0.06	0.39	1535	0.93 (0.75 -1.16)	0.52	0.79	genotyped
rs7861779	*RXRA*	2766	1.27 (0.97 -1.65)	0.08	0.40	1536	1.29 (1.00 -1.65)	**0.049**	0.35	imputed
rs3118538	*RXRA*	2829	1.80 (0.57 -5.70)	0.32	0.61					N/A
rs1151	*PPP1R14*	2681	1.07 (0.83 -1.37)	0.62	0.87	1536	1.02 (0.80 -1.30)	0.87	0.93	genotyped
rs3829251	*NADSYN1*	2791	1.04 (0.76 -1.43)	0.81	0.93	1536	1.34 (1.06 -1.69)	**0.016**	0.25	genotyped
rs7041	*GC*	2785	0.77 (0.63 -0.94)	**0.012**	0.22	1536	1.13 (0.94 -1.35)	0.19	0.54	genotyped
rs222040	*GC*	3382	0.80 (0.67 -0.96)	**0.016**	0.22	1536	0.91 (0.76 -1.09)	0.30	0.65	imputed
rs12512631	*GC*	2788	1.19 (0.96 -1.48)	0.10	0.40	1536	0.85 (0.70 -1.03)	0.098	0.45	genotyped
rs2282679	*GC*	2794	0.97 (0.77 -1.23)	0.81	0.93	1536	1.02 (0.84 -1.24)	0.86	0.93	genotyped
rs1790349	*DHCR7*	2811	1.04 (0.77 -1.40)	0.79	0.93	1536	1.37 (1.08 -1.73)	**0.009**	0.25	genotyped
rs3787555	*CYP2R1*	2788	1.25 (1.01 -1.56)	**0.042**	0.33	1535	0.94 (0.75 -1.18)	0.59	0.79	genotyped
rs4809960	*CYP2R1*	2731	1.15 (0.91 -1.45)	0.25	0.54	1536	0.84 (0.68 -1.04)	0.10	0.45	genotyped
rs2244719	*CYP2R1*	2715	1.17 (0.88 -1.57)	0.29	0.59					N/A
rs2762939	*CYP2R1*	2759	1.11 (0.86 -1.45)	0.42	0.71	1536	1.11 (0.89 -1.38)	0.38	0.77	imputed
rs2296241	*CYP2R1*	2784	0.94 (0.76 -1.16)	0.55	0.82	1536	1.15 (0.96 -1.39)	0.13	0.45	genotyped
rs927650	*CYP2R1*	2770	1.06 (0.86 -1.30)	0.60	0.86	1536	1.06 (0.88 -1.27)	0.52	0.79	genotyped
rs2181874	*CYP2R1*	2792	1.04 (0.83 -1.32)	0.73	0.93	1536	1.07 (0.86 -1.33)	0.55	0.79	genotyped
rs4809959	*CYP2R1*	2756	0.97 (0.79 -1.20)	0.79	0.93	1536	1.21 (1.01 -1.45)	**0.043**	0.35	genotyped
rs2060793	*CYP2R1*	2788	0.98 (0.80 -1.21)	0.88	0.94	1536	1.02 (0.85 -1.23)	0.84	0.93	genotyped
rs2762941	*CYP2R1*	2802	0.99 (0.80 -1.22)	0.90	0.95	1534	1.13 (0.93 -1.38)	0.22	0.56	genotyped
rs6022999	*CYP2R1*	2811	1.01 (0.79 -1.29)	0.92	0.95	1536	0.89 (0.71 -1.11)	0.29	0.64	genotyped
rs4646536	*CYP27B1*	2799	1.02 (0.82 -1.27)	0.83	0.94	1536	0.91 (0.75 -1.11)	0.36	0.76	imputed
rs7594289	*CYP27A1*	2747	0.97 (0.79 -1.18)	0.72	0.93	1536	1.26 (1.05 -1.52)	**0.012**	0.25	imputed
rs35456792	*CDKN1B*	2819	0.00 (0.00- >999)	0.97	0.97					N/A
rs1801270	*CDKN1A*	2789	1.14 (0.84 -1.54)	0.39	0.70	1536	1.03 (0.71 -1.50)	0.88	0.93	imputed
rs1059234	*CDKN1A*	3426	1.06 (0.75 -1.50)	0.76	0.93	1536	1.03 (0.71 -1.50)	0.88	0.93	imputed
rs3218089	*CCND3*	2762	0.65 (0.38 -1.10)	0.11	0.40					N/A
rs1051130	*CCND3*	2781	0.98 (0.79 -1.22)	0.86	0.94	1536	1.04 (0.86 -1.25)	0.71	0.89	imputed
rs6599638	*C10orf88*	2754	1.14 (0.93 -1.41)	0.22	0.50	1536	1.00 (0.83 -1.19)	0.97	0.98	genotyped

^a^ GEM study: model adjusted for age, sex, center, status, time-dependent crossover status, and log transformed Breslow thickness; HR (95% CI): per minor allele hazard ratio (95% confidence interval).

^b^ MD Anderson study: model adjusted for age, sex, and log transformed Breslow thickness; HR (95% CI): per minor allele hazard ratio (95% confidence interval).

^c^ The SNP was not genotyped/imputed in the MD Anderson study.

## Discussion

Utilizing the population-based GEM study, we did not observe an enhanced prognostic classification for melanoma by incorporating VitD pathway SNPs into the known major prognostic measures (i.e. age, sex, Breslow thickness, mitoses and ulceration). Using the MD Anderson study as an independent validation, we observed similar results that Breslow thickness, ulceration, mitoses and age are consistently selected as the top prognostic variables. When additional prognostic factors (i.e. histology, site, sun exposure, and phenotypic index) were included in the analysis of the GEM study, the tumor factors (histology and site) were again selected as variables with higher prognostic effects compared to VitD pathway SNPs.

Findings using a sophisticated random survival forest (RSF) approach and Cox proportional hazards model and application to two independent melanoma studies yield similar results. While both RSF and Cox models aim to identify variables that best predict the survival outcome, the mechanisms implemented in the two algorithms are different. The Cox regression model is the widely used method for investigating the relationship between covariates of interest and survival outcome, which estimates the log-linear relationship between covariates and the underlying hazard function and provides clinically interpretable results. The RSF method is a nonparametric machine learning method which assesses the prediction accuracy of ensemble trees, and introduced two randomnesses where a randomly drawn bootstrap sample was used to grow a tree and at each split a randomly selected subset of variables was selected as candidates [15, 20]. The RSF method was demonstrated to outperform the Cox model when possible non-linear relationships exist [21]. Due to the aforementioned different mechanisms used in the two methods, we observed that SNPs with the highest VIMP values may not necessarily be those identified in Cox regression model. However, the overall conclusion that VitD pathway SNPs do not provide significant prognostic improvement for melanoma over current prognostic system is consistent in both analyses.

Although the two independent studies have different designs, population-based vs. hospital-based, and thus have distinct patient characteristics, we consistently observed higher prognostic effects for the clinical factors known to be associated with melanoma outcome compared to the SNPs in predicting melanoma specific survival. This lack of predictability suggests the limited potential use of adding VitD pathway SNPs to the prognostic system for melanoma. Studies investigating the improvement of including genetic factors in predicting melanoma risk have reported effects with a variety of magnitude. It was reported that the improvement of melanoma risk prediction by adding *MC1R* to age, sex, and cutaneous melanin phenotypes is modest and too small to be valuable in clinical setting [22]. Other studies have reported statistically significant, but small [23] to modest [24] improvement in prediction of melanoma risk by adding *MC1R* genotype to traditional demographic and pigmentation characteristics.

It is important to note from our power analysis that the GEM study has a sufficiently large sample size to detect increase in AUC. In the GEM study sample, we have 92% power to detect an increase of 0.04 between a diagnostic test with an AUC of 0.80 and another diagnostic test with an AUC of 0.84 using a one-sided z-test at a significance level of 0.05. The modest improvement in prognostic effects by including the VitD pathway SNPs is not likely due to the sample size issue.

The large-scale genotyping technologies and genetic epidemiology studies of melanoma provide promise for unraveling patients’ genetic makeup and developing genetic prognostic markers for melanoma progression. The VitD pathway SNPs were reported in previous publications to be significantly associated with melanoma specific survival [3, 4], suggesting its key important role in understanding the biological mechanisms for melanoma progression. To date there are limited evidence for SNPs that significantly predict melanoma survival, and the reported effect sizes of SNP predictability in survival are typically small [25, 26]. The findings from this study yielded similar results. We did not find an improvement in melanoma prognosis beyond that attributed to known prognostic variables by including VitD pathway SNPs. The genetic variations contributing to melanoma survival and progression are likely to be multi-dimensional, and may involve complicated biological pathway functionality and gene environment interactions in addition to single SNPs and warrants further investigation.

Besides the strengths of this study, we have noted some limitations. The major limitation of our study is that instead of a genome-wide association study investigating all human genes, we have conducted a confirmatory study which focused on a subset of SNPs in the vitamin D Pathway that were previously reported to be associated with melanoma or biologically functionally relevant. Second, other than the classical vitamin D pathway, we have not investigated the recently reported novel alternative pathways of vitamin D activation which may have complicated the findings. Different from classical vitamin D activation, novel pathways of vitamin D3 and 7-dehydrocholesterol initiated by *CYP11A1* were recently reported [27], which were demonstrated to be of significant physiological role [28]. Novel vitamin D3 hydroxyderivatives resulting from *CYP11A1* action were detectable in human epidermis and serum and in pig adrenal glands [29]. It was demonstrated that the endogenously produced novel D3 hydroxyderivatives can act as biased agonists of VDR and inverse agonists of the retinoic acid-related orphan receptors (RORα and RORγ) [30, 31]. It was also shown that RORα and RORγ are expressed in normal and pathological human skin [30], and that decreased expression of RORα and RORγ is associated with the development and progression of melanoma [32]. Third, we had limited ability to explore some factors previously demonstrated to contribute to melanoma prognosis. The reduced expression of vitamin D receptor (*VDR*) was reported to be related to shorter overall survival [33]. Low expression of the Vitamin D activating enzyme 1α-Hydroxylase (*CYP27B1*) were found to be associated with shorter overall survival and disease free survival in melanomas[34]. Fourth, we acknowledge the limitation that we have not collected data on patients’ actual vitamin D status such as blood level of 25-hydroxyvitamin D and 1, 25-dihydroxyvitamin D. It would be important to evaluate the role of actual vitamin D status on melanoma prognosis that potentially confounds the SNP effects, which may provide more information and shed light on clinical decision making, such as vitamin D supplement for melanoma patients based on their genetic background.

Future larger genome-wide or sequencing studies exploring the melanoma prognostic effects more comprehensively by including SNPs from alternative pathways of vitamin D activation as well as other biological pathways are suggested. Evaluation of the association between actual vitamin D status and melanoma survival that are independent of SNPs would also contribute to our understanding of the melanoma prognosis. Finally, in terms of the biology we may find more meaningful results when accounting for sun exposure, and therefore our future work will include investigating the prognostic effects of gene-environment interactions. We will also explore combining the prognostic effects of SNPs into that of biologically meaningful genes or pathways, and evaluate the improvement of melanoma prognosis by adding genes or pathways in the future.

## Supporting information

S1 Fig A diagram of genes involved in the Vitamin D pathway adapted from a previous report [11]. (PNG)

S1 Table The list of SNPs along with their chromosome locations, minor/major alleles in the population, gene names, minor allele frequency (MAF) in the GEM study participants, and genotyping platform. (DOCX)
