# Supplementary figures and images for: No prognostic value added by vitamin D pathway SNPs to current prognostic system for melanoma survival
Source: PLoS One. 2017 Mar 21;12(3):e0174234. doi: 10.1371/journal.pone.0174234 (PMC5360355; doi:10.1371/journal.pone.0174234)

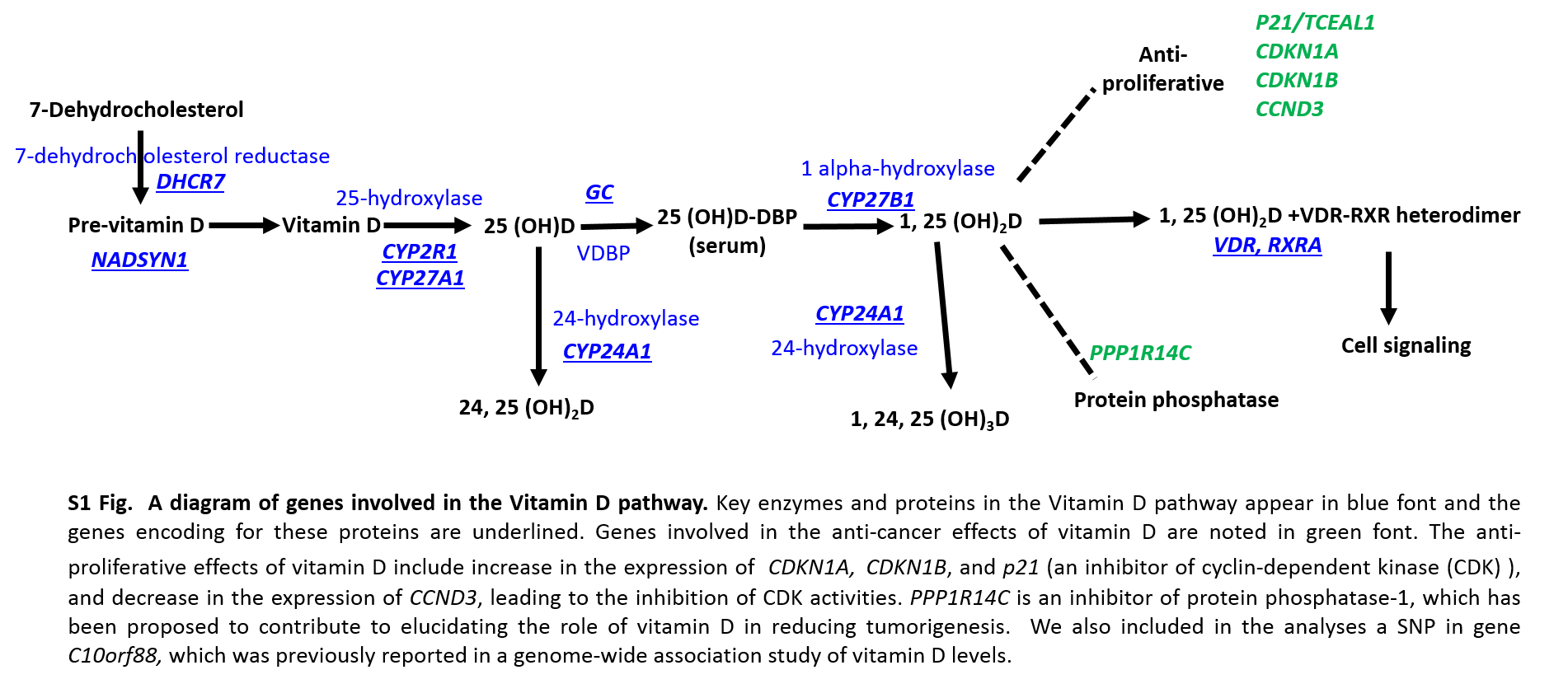

Supplement: S1 Fig — (PNG) [file pone.0174234.s001.png]
