# Supplementary material for: No prognostic value added by vitamin D pathway SNPs to current prognostic system for melanoma survival
Source: PLoS One. 2017 Mar 21;12(3):e0174234. doi: 10.1371/journal.pone.0174234 (PMC5360355; doi:10.1371/journal.pone.0174234)
Supplement: S1 Table — (DOCX) [file pone.0174234.s002.docx]

S1 Table. The list of SNPs included in the analyses.

| **snp** | **gene** | **minor** | **major** | **chromosome** | **Position (hg19)** | **MAF** | **Platform** |
| --- | --- | --- | --- | --- | --- | --- | --- |
| rs2544038 | *VDR* | C | T | 12 | 48215233 | 0.449 | Sequenom |
| rs2544028 | *VDR* | A | T | 12 | 48216430 | 0.417 | Sequenom |
| rs2544027 | *VDR* | T | C | 12 | 48216529 | 0.488 | Sequenom |
| rs7965281 | *VDR* | A | G | 12 | 48231610 | 0.482 | Sequenom |
| rs11574143 | *VDR* | A | G | 12 | 48234917 | 0.108 | Illumina |
| rs11574139 | *VDR* | T | A | 12 | 48235555 | 0.038 | Sequenom |
| rs731236 | *VDR* | C | T | 12 | 48238757 | 0.405 | Sequenom |
| rs1544410 | *VDR* | A | G | 12 | 48239835 | 0.403 | Pyrosequencing |
| rs7305032 | *VDR* | G | A | 12 | 48249860 | 0.460 | Sequenom |
| rs2238140 | *VDR* | C | T | 12 | 48252664 | 0.488 | Sequenom |
| rs12370156 | *VDR* | C | T | 12 | 48254133 | 0.488 | Sequenom |
| rs2239182 | *VDR* | A | G | 12 | 48255411 | 0.478 | Sequenom |
| rs2107301 | *VDR* | T | C | 12 | 48255570 | 0.276 | Sequenom |
| rs2239181 | *VDR* | G | T | 12 | 48255949 | 0.104 | Sequenom |
| rs886441 | *VDR* | C | T | 12 | 48262964 | 0.195 | Sequenom |
| rs2189480 | *VDR* | A | C | 12 | 48263828 | 0.359 | Sequenom |
| rs3782905 | *VDR* | G | C | 12 | 48266167 | 0.325 | Sequenom |
| rs7974708 | *VDR* | C | T | 12 | 48270165 | 0.349 | Sequenom |
| rs11168275 | *VDR* | G | A | 12 | 48272275 | 0.246 | Sequenom |
| rs2228570 | *VDR* | T | C | 12 | 48272895 | 0.391 | Melting temperature |
| rs2254210 | *VDR* | A | G | 12 | 48273714 | 0.363 | Illumina |
| rs1989969 | *VDR* | T | C | 12 | 48278010 | 0.397 | Sequenom |
| rs2238135 | *VDR* | C | G | 12 | 48278190 | 0.244 | Sequenom |
| rs4760648 | *VDR* | T | C | 12 | 48280665 | 0.434 | Sequenom |
| rs10875694 | *VDR* | A | T | 12 | 48281660 | 0.170 | Sequenom |
| rs11168284 | *VDR* | G | A | 12 | 48283049 | 0.356 | Sequenom |
| rs11168287 | *VDR* | A | G | 12 | 48285414 | 0.498 | Sequenom |
| rs7299460 | *VDR* | T | C | 12 | 48296268 | 0.303 | Sequenom |
| rs4516035 | *VDR* | C | T | 12 | 48299826 | 0.432 | Sequenom |
| rs7139166 | *VDR* | G | C | 12 | 48300334 | 0.431 | Sequenom |
| rs11568820 | *VDR* | A | G | 12 | 48302545 | 0.175 | Sequenom |
| rs10459217 | *VDR* | C | T | 12 | 48316261 | 0.206 | Sequenom |
| rs11168314 | *VDR* | A | G | 12 | 48330629 | 0.200 | Sequenom |
| rs4073729 | *VDR* | T | C | 12 | 48337069 | 0.148 | Sequenom |
| rs4237856 | *VDR* | C | A | 12 | 48338050 | 0.248 | Sequenom |
| rs1015390 | *VDR* | T | C | 12 | 48344038 | 0.146 | Sequenom |
| rs4760674 | *VDR* | A | C | 12 | 48357014 | 0.385 | Sequenom |
| rs6823 | *VDR* | G | C | 12 | 48362412 | 0.448 | Sequenom |
| rs10875712 | *VDR* | C | G | 12 | 48363253 | 0.368 | Sequenom |
| rs2071358 | *VDR* | A | C | 12 | 48366449 | 0.171 | Sequenom |
| rs34421776 | *TCEAL1* | A | G | X | 102884857 | 0.006 | Illumina |
| rs10776909 | *RXRA* | A | G | 9 | 137288746 | 0.302 | Illumina |
| rs7861779 | *RXRA* | A | G | 9 | 137309461 | 0.149 | Illumina |
| rs3118538 | *RXRA* | A | G | 9 | 137460465 | 0.002 | Illumina |
| rs1151 | *PPP1R14* | C | A | 6 | 150571265 | 0.234 | Illumina |
| rs3829251 | *NADSYN1* | A | G | 11 | 71194559 | 0.130 | Illumina |
| rs12512631 | *GC* | G | A | 4 | 72601331 | 0.358 | Illumina |
| rs2282679 | *GC* | C | A | 4 | 72608383 | 0.292 | Illumina |
| rs222040 | *GC* | C | T | 4 | 72616932 | 0.430 | Sequenom |
| rs7041 | *GC* | A | C | 4 | 72618334 | 0.433 | Illumina |
| rs1790349 | *DHCR7* | G | A | 11 | 71142350 | 0.138 | Illumina |
| rs2060793 | *CYP2R1* | A | G | 11 | 14915310 | 0.390 | Illumina |
| rs927650 | *CYP2R1* | A | G | 20 | 52772741 | 0.485 | Illumina |
| rs2762939 | *CYP2R1* | G | C | 20 | 52781251 | 0.182 | Illumina |
| rs3787555 | *CYP2R1* | A | C | 20 | 52782691 | 0.262 | Illumina |
| rs2244719 | *CYP2R1* | A | G | 20 | 52782858 | 0.396 | Illumina |
| rs2762941 | *CYP2R1* | A | G | 20 | 52783652 | 0.372 | Illumina |
| rs2181874 | *CYP2R1* | A | G | 20 | 52784478 | 0.232 | Illumina |
| rs4809959 | *CYP2R1* | A | G | 20 | 52785859 | 0.480 | Illumina |
| rs4809960 | *CYP2R1* | G | A | 20 | 52786073 | 0.266 | Illumina |
| rs2296241 | *CYP2R1* | G | A | 20 | 52786219 | 0.477 | Illumina |
| rs6022999 | *CYP2R1* | G | A | 20 | 52788013 | 0.236 | Illumina |
| rs4646536 | *CYP27B1* | G | A | 12 | 58157988 | 0.318 | Illumina |
| rs7594289 | *CYP27A1* | G | A | 2 | 219652286 | 0.452 | Illumina |
| rs35456792 | *CDKN1B* | A | G | 12 | 12870941 | 0.003 | Illumina |
| rs1801270 | *CDKN1A* | A | C | 6 | 36651971 | 0.096 | Illumina |
| rs1059234 | *CDKN1A* | T | C | 6 | 36653597 | 0.067 | Sequenom |
| rs1051130 | *CCND3* | A | C | 6 | 41903782 | 0.436 | Illumina |
| rs3218089 | *CCND3* | A | G | 6 | 41908122 | 0.060 | Illumina |
| rs6599638 | *C10orf88* (vicinity of gene *ACADSB* ) | A | G | 10 | 124704149 | 0.494 | Illumina |
